# Parental and Demographic Predictors of Engagement in an mHealth Intervention: Observational Study From the Let’s Grow Trial

**DOI:** 10.2196/60478

**Published:** 2025-07-15

**Authors:** Johanna Sandborg, Brittany Reese Markides, Savannah Simmons, Katherine L Downing, Jan M Nicholson, Liliana Orellana, Harriet Koorts, Valerie Carson, Jo Salmon, Kylie D Hesketh

**Affiliations:** 1 Institute for Physical Activity and Nutrition (IPAN) Faculty of Health Deakin University Geelong, Victoria Australia; 2 Judith Lumley Centre La Trobe University Bundoora Australia; 3 Faculty of Health Biostatistics Unit Deakin University Geelong Australia; 4 Faculty of Kinesiology, Sport, and Recreation University of Alberta Edmonton, AB Canada

**Keywords:** digital intervention, parenting, movement behaviors, sleep, screen time, early childhood

## Abstract

**Background:**

Parents are integral in shaping early childhood health behaviors, and mobile health (mHealth) interventions offer an accessible method of supporting them in this role. Optimizing participant engagement is key to mHealth effectiveness and impact; however, research examining personal predictors of engagement remains underexplored.

**Objective:**

We aimed to describe participant engagement with a novel parental mHealth intervention (Let’s Grow) during the first 25 weeks of use and investigate whether engagement levels varied by family demographics and parental cognitions and behaviors relevant to the intervention.

**Methods:**

We used data from parents in the intervention group of the Let’s Grow trial. The intervention targeted toddlers’ movement behaviors, and the program (a purpose-designed progressive web app) was delivered via self-paced modules. The content was built around 3 main components (behavior change activities, information provision, and social support). Engagement data (web app analytics) collected across the first 25 weeks of the intervention were summarized as study-specific metrics (time using the app, proportion of accessed features and pages, clicks in the main parts of the app) and overall engagement measures (composite engagement index [EI], individual subindexes [click depth, loyalty, recency, and diversity]). The baseline measures included family demographics (main carer, child and family characteristics, and postcode) and parental cognitions and behaviors relevant to the intervention (coping, concern, and information seeking). Linear regression was used to assess associations between baseline and engagement measures.

**Results:**

All parents allocated to the intervention group (n=682) were included. Most parents (609/682, 89.3%) logged in and used at least 1 app feature; those who never used the app were excluded from subsequent analyses. App access declined from 90.6% (552/609) in the first week to 31.2% (190/609) at 25 weeks. For users active during weeks 12 to 25, EI remained consistent and was nearly identical to the average EI (28%, range 3%-50%). More work hours, parents living together, having siblings in the family, and living in a regional or remote area were each associated with lower engagement on 10 out of 12 indicators (β=−31.37 to −0.01; all *P*≤.046). Higher education level was associated with higher engagement on 9 indicators (β=0.77-18.59; all *P*≤.02). Of the parental characteristics, only higher coping was positively associated with engagement (β=1.25; *P*=.003).

**Conclusions:**

Our findings indicate that time and sociodemographic factors might be the most relevant predictors of engagement and highlight the characteristics of parents who may benefit from more active strategies to support their engagement with digital interventions. The uptake and continued engagement with this app exceeded what is generally reported for apps, but it is unknown whether this is sufficient for behavior change. Individual and composite engagement measures yielded similar results, indicating that simpler, more feasible metrics can be useful for reporting engagement in digital interventions.

**International Registered Report Identifier (IRRID):**

RR2-10.1136/bmjopen-2021-057521

## Introduction

### Background

Early childhood (age 0-5 years) has been identified as a key time to promote healthy behaviors (eg, physical activity) [1]. During these formative years, parents play an important role in providing opportunities for their children to develop these behaviors, and parental encouragement and support have been shown to positively influence children’s health behaviors [2,3]. Parental involvement in childhood interventions has also been shown to be effective in promoting a healthy diet and physical activity in children [4], with greater effects from interventions that include parenting skill training and behavior change strategies [5]. Mobile health (mHealth) interventions (eg, mobile apps, websites, or messaging services) offer a convenient and accessible way to provide support and timely information to parents and can enable participation regardless of geographic location. This delivery mode has been shown to be well accepted among parents and effective in promoting health behaviors in children [6-8].

The digital format of mHealth interventions also enables the use of built-in tools for the objective measurement of user engagement (ie, *how* and *to what extent* users engage with the intervention), which is directly linked to intervention effectiveness [9]. Examining user engagement is a critical component of evaluating mHealth interventions as it provides information on levels of intervention exposure and uptake and can facilitate the identification of factors that may optimize engagement [10]. Understanding who and how participants engage with digital interventions can also provide insight into the mechanisms that drive intervention effectiveness and contribute to the design of more impactful interventions. However, there is a notable lack of reporting on engagement and its predictors in mHealth interventions targeting parents of young children, with only a few studies (eg, [11-15]) presenting parent engagement using objective data rather than relying on parental reports. Among the studies that have investigated predictors of engagement in mHealth parental interventions [11-14], only family demographics (parental education [11-14], number of children in the household [11,13], and work status [12]) have been examined. No previous studies have investigated parental cognitions and behaviors despite evidence that parental constructs, such as parental confidence, can predict dropout rate in traditional family-based interventions [16]. Thus, little is known about how parents of young children engage with mHealth interventions or about the potential influence that family demographics and parental cognitions and behaviors may have on parents’ engagement with these interventions.

Let’s Grow is a novel mHealth intervention designed to support parents in improving the movement behaviors (ie, physical activity, sedentary behavior, and sleep) of their children aged 2 years via a purpose-built app [17]. The program is delivered via modules that parents work through over time, and the content is built around 3 main components, that is, behavior change activities, information provision, and social support, all of which have been identified as key strategies to promote health behaviors [18].

### Objectives

To the best of our knowledge, no previous study has comprehensively investigated engagement and predictors of engagement in a parental mHealth intervention. Thus, we aimed to use data from the Let’s Grow trial [17] to (1) describe parental engagement with the Let’s Grow app during the first 25 weeks of use and (2) investigate whether engagement levels varied by family demographics and parental cognitions and behaviors relevant to the intervention (hereafter referred to as parental characteristics).

## Methods

### Study Design

We used data from the Let’s Grow randomized controlled trial (ACTRN12620001280998; U1111-1252-0599) [17] in this observational study. The trial design and rationale have been described previously [17]. In brief, the trial investigated a 12-month mHealth intervention (delivered through a progressive web app, the Let’s Grow app) aimed at supporting parents of children aged 2 years to improve their child’s movement behaviors [17]. This study is reported according to the STROBE (Strengthening the Reporting of Observational Studies in Epidemiology) checklist.

### Ethical Considerations

The Let’s Grow trial received ethics approval from the Deakin University Human Research Ethics Committee, Australia (2020-077). The main carer was provided with details of the study commitment before providing consent for their own participation and that of their child via an online form. Participants received an Aus $20 (US $13) retail gift voucher upon completion of the study assessments (survey and activity monitors) at each time point; no compensation was provided for engaging with the app. All participant data were deidentified and securely stored in accordance with the Deakin University protocols.

### Recruitment, Randomization, and Procedures

Participants were recruited through social media (eg, Facebook and parenting blogs) between March 2021 and June 2022. Parents aged ≥18 years, residing in Australia, owning a mobile phone, having internet access, literate in English, and with a child aged 18 to 35 months walking independently were eligible. Following baseline assessment, participants were randomized in a 1:1 ratio stratified by geographical location (urban, or outer regional and remote), yielding 16 strata across the 8 Australian states and territories. The intervention group (n=682) was then provided access to the Let’s Grow app. The baseline survey data and app analytics metrics of the intervention group participants collected during the first 25 weeks of app access were used for this study.

### The Let’s Grow App

The development and content of the Let’s Grow app have been described in detail elsewhere [17]. In brief, the app was built around 3 main components, including active learning targeting changes in specific combinations of movement behaviors (learning modules), general information provision (toolkit), and social support (community chat forum) to help parents improve their child’s physical activity, sedentary behavior (including screen time), and sleep. The active learning component consisted of 8 modules: “Switch off and play,” “Play, sleep, repeat,” “Avoid light sleep tight,” “Play skills for life,” “Build your best day,” “Parents provide, kids decide,” “Rocking routines,” and “Calm families”. Each module contained information and strategies (in text, pictorial, podcast, and video format) on child sleep, play, screen time, and general parenting, as well as a set of behavior change activities, including both online and offline components. The modules were not mutually exclusive to 1 behavior as all modules covered parenting and behavior change strategies for at least 2 out of the 3 target child behaviors, with a focus on the interplay between child movement behaviors (eg, increasing physical activity to decrease sedentary time). To further support parents, each module also included SMS text messaging (eg, interactive messages that facilitate goal setting and self-monitoring).

While the parents could complete the modules in any order, they were only able to access 1 module at a time and were required to complete all activities within that module before moving on to another. As activities took varying amounts of time to complete (eg, self-monitoring occurred across a week), the minimum amount of time to complete a module before moving on to the next ranged from 2 to 5 weeks. The completed modules remained accessible for the participants to revisit information. However, if they did not complete a module, the information contained in the other modules would be inaccessible to them. The intervention duration was 12 months; the minimum timeframe to complete all the 8 modules was 22 weeks, but participants were not expected to complete all the modules within this timeframe. Upon completion of all the modules, the participants received SMS text messaging notifications once a week to refresh their knowledge and encourage revisiting of the app. The toolkit and community chat forum remained freely accessible throughout the intervention period. The toolkit included resources such as outdoor and indoor play ideas, screen-free activities, a podcast providing help with children’s common sleep issues, and videos explaining different parenting strategies in relation to managing difficult child behavior. The forum provided the parents with the opportunity to anonymously share ideas and connect with other participants. The parents were prompted with SMS text message reminders to reengage with the app if no activity was detected for an extended period (eg, if the app had not been opened for 3 weeks). An overview with screenshots of the Let’s Grow app is presented in Multimedia Appendix 1.

### Factors Related to Engagement

At baseline, the main carer completed an online survey (using REDCap [Research Electronic Data Capture; Vanderbilt University]) capturing family demographics and parental characteristics. Main carer characteristics considered in this study included age (years), relationship with the child (mother or father), birth country (Australian-born, yes or no), education level (university degree vs no university degree), and work status (currently working, yes or no). Participant child characteristics included sex and temperament in comparison to other children (single item; scale 1-5 ranging from much easier than average to much more difficult than average) [19]. Family characteristics included parents living together (yes or no) and siblings in the family (yes or no). Postcode was used to derive information on remoteness, that is, major cities versus inner or outer regional and rural Australia, which was based on the Australian Bureau of Statistics Australian Statistical Geography Standard Remoteness Structure [20], as well as area-level socioeconomic status derived using the Australian Bureau of Statistics Socio-Economic Indexes for Areas Index of Relative Socio-economic Advantage and Disadvantage [21].

Three parental characteristics relevant to the intervention were considered: parent coping in general, concern about the targeted topic (movement behaviors), and information seeking related to the targeted topic. Parents reported using a 5-point scale (1=not at all, 5=extremely well, possible range 1-5) how they were coping with life at present [22]. In terms of concern with child movement behaviors, the parents reported about their child’s sitting time, physical activity or active play, screen use, and sleep to indicate whether they considered it a problem using a 5-point scale (1=not a problem at all to 5=a serious problem) [23]. A combined movement behavior concern score was calculated as the sum of the individual scores (possible range 4-20; higher scores indicating greater concern) [23]. Finally, the parents reported how much time in a usual week they spend using apps or websites to access information on children’s physical activity, sitting time or screen use, and sleep, respectively. The total reported time searching for information on child movement behaviors and child health topics was calculated (h per wk; possible range 0-40 h per wk).

### Metrics of Engagement

Data on users’ activities and actions in the Let’s Grow app were logged to a dedicated database connected to the app’s backend system. These data were used to report user engagement descriptively, such as the proportion of participants who initiated app use (ie, intervention uptake), the types of features they used (ie, learning modules, toolkit, and community chat forum), the number of modules they completed, the total time spent in the app (min), the total days of app use, the proportion of pages (%) and features (%) visited at least once throughout the 25 weeks, and the number of actions (posts, likes, and comments) in the community chat forum.

To assess overall engagement among the Let’s Grow app users, an adapted version of a previously developed engagement index (EI) [11,24] was calculated. This index assessed different aspects of engagement compared to the descriptive engagement measures and is more broadly applicable across different apps. As per previous studies [11,14,24], the 4 subindexes relevant to this program, from the original 7 in the index, were used. These included the following: (1) click depth (average number of app pages viewed per day); (2) loyalty (total days with any use data for a participant); (3) recency (average number of days between app use; reversed so a higher score indicates greater engagement); and (4) diversity (the number of different app features used per day, ie, activities within modules, toolkit, community chat forum, and frequently asked questions). A detailed description of the individual subindexes is presented in Multimedia Appendix 2. The subindexes were normalized by rescaling values between 0% and 100% to assign equal weight to each of the subindexes. The EI was calculated as the average of the 4 subindexes. In addition, a weekly EI (range 0%-100%) was calculated for those with engagement data in any particular week to enable a more granular investigation into participant engagement over the 25 weeks. All calculations were carried out using Python (version 3; Python Software Foundation).

### Covariates

The Let’s Grow trial was conducted during the COVID-19 pandemic (baseline data collection from March 2021 to June 2022; the data for this paper were collected up to January 2023). There were varying pandemic restrictions across different states and territories in Australia, which potentially impacted the everyday life of the participating families and their ability to engage with the program. Thus, the data on the number of days in lockdown during the 6-month engagement period were calculated based on individual postcode and publicly available data on local restrictions. In addition, due to technical issues, the app was not functioning at certain periods; nevertheless, due to the staggered recruitment, not all participants experienced equal app downtime, which might have differentially affected the engagement. The app downtime was calculated for each participant using web app analytics.

### Data Analysis

All statistical analyses were carried out using the statistical software R (version 4.0.3; R Foundation for Statistical Computing). Chi-square tests for categorical and *t* test for numerical variables were used to investigate differences between those who logged in at least once and used at least 1 app feature (ie, users: 609/682, 89.3%) and those who never logged in or logged in but never used an app feature (ie, nonusers: 73/682, 10.7%). Further analyses involved only the app users. The associations between the potential predictors (family demographics and parental characteristics) and the individual (total time spent in the app and clicks in the main parts of the app: learning modules, toolkit, and community chat forum) and the overall measures of engagement (EI and subindexes) at 25 weeks were estimated using linear regression. Sensitivity analyses were performed to adjust for the lockdown and app downtime by including the number of days in lockdown and the total app downtime (days) in the models. Estimates from the sensitivity analyses are presented alongside the main analyses where results differed.

## Results

### Participant Characteristics

The baseline characteristics of the 682 participants randomized to the intervention group are reported in Table 1. The participants were, on average, aged 34.9 (SD 4.5; range 21-48) years; the majority were mothers (670/682, 98.2%), Australian-born (522/681, 76.7%), university educated (490/682, 71.8%), lived in major cities (490/682, 71.8%), and lived together with a partner or spouse (629/681, 92.4%). In terms of intervention uptake, 67 (9.8%) of the 682 participants never logged in, and a further 6 (0.9%) did not progress past the log-in page to use any of the app features. Of the 682 participants, the 609 (89.3%) who logged in at least once and used at least 1 app feature were referred to as *users* and were included in the subsequent analyses. The users did not differ from the nonusers in terms of the potential predictors of engagement at baseline, with the exception of reporting a higher coping score (mean score 3.2 vs 3.0; *P*=.01) and a higher level of concern with their child’s movement behaviors (mean score 7.4 vs 6.8; *P*=.04).

**Table 1:** Family demographics and parental characteristics of the participants in the intervention group of the Let’s Grow trial (n=682)^a^.

Family demographics and parental characteristics				Values
**Parent^b^**				
	Age (y), mean (SD)			34.9 (4.5)
	Mother, n (%)			670 (98.2)
	Australian-born, n (%)			522 (76.7)
	University degree, n (%)			490 (71.8)
	Currently working, n (%)			442 (64.9)
	Parental coping, mean (SD)			3.2 (0.7)
	Concern with their child’s movement behaviors, mean (SD)			7.3 (2.6)
	Information seeking (h/wk), mean (SD)			2.2 (4.6)
**Child**				
	Female, n (%)			337 (49.4)
	**Child temperament, n (%)**			
		Much easier or easier	221 (32.4)	
		Average	361 (52.9)	
		More or much more difficult	100 (14.7)	
**Family, n (%)**				
	Living with a partner or spouse			629 (92.4)
	Siblings in the family			361 (58.8)
**Living area and area-level socioeconomic status**				
	**Remoteness level, n (%)**			
		Major cities	490 (71.8)	
		Inner regional Australia	131 (19.2)	
		Outer regional or remote Australia	61 (8.9)	
	**Socio-Economic Indexes for Areas category, n (%)**			
		Low advantaged/high disadvantaged	136 (20)	
		Middle advantaged/disadvantaged	223 (32.8)	
		High advantaged/low disadvantaged	321 (47.2)	

^a^Due to missing data, n varied slightly for some variables, with missing data for individual variables ranging from n=0 to n=68.

^b^These data are from the self-selected main carer of the child.

### User Engagement With the Let’s Grow App

Table 2 presents the different measures of engagement for those classified as users, including overall use and engagement with the different components of the app. Across the 25 weeks, users spent an average of 31 (SD 36) minutes in the app and accessed the app on an average of 18 (SD 15) separate days over this time. The users visited on average, 13% (SD 13%) of the pages and 40% (SD 18%) of the app features, noting that a large proportion of the content was contained within the learning modules and, thus, remained inaccessible until the modules were completed.

**Table 2:** Objective measures of the app engagement after 6 months in the participants defined as app users (n=609).

Engagement metrics					Values
**Individual engagement metrics**					
	**Overall use**				
		Total time spent in the app (min), mean (SD; range)		31 (36; 0-270)	
		App use (d), mean (SD; range)		18 (15; 1-82)	
		**Proportion of content viewed (%; range)**			
			Pages visited	12.8 (13; 1-77)	
			Features visited	39.6 (18.5; 5-100)	
	**Engagement in the main parts of the app**				
		**At least 1 time access, n (%)**			
			Learning modules	505 (82.9)	
			Toolkit	335 (55)	
			Community chat forum	177 (29.1)	
		**Clicks (n), mean (SD; range)**			
			Learning modules	58.8 (74.8; 0-497)	
			Toolkit	17.6 (40.2; 0-356)	
			Community chat forum	1.8 (4.8; 0-42)	
**Composite engagement metrics**					
	Total engagement index, mean (SD; range)			28 (6.6; 3.2-50)	
	**Subindexes, mean (SD; range)**				
		Click depth		2.4 (2.4; 0.1-17.8)	
		Loyalty		9.2 (7.6; 0-46.4)	
		Recency		87.4 (14.9; 0-100)	
		Diversity		13 (9.5; 0-66.7)	

The highest level of engagement was observed in the learning modules, with 82.9% (505/609) of the users accessing the modules at least once. The average number of completed modules was 2.4 (SD 3.0; range 0-8) out of the possible 8. More than half of the users (329/609, 54%) completed at least 1 module: 18.1% (110/609) completed 1 module, 8.2% (50/609) completed 2 modules, 7.6% (46/609) completed 3 modules, 6.4% (39/609) completed 4 modules, 6.7% (41/609) completed 5 modules, 3.9% (24/609) completed 6 modules, 2.1% (13/609) completed 7 modules, and 1% (6/609) completed all 8 modules by 25 weeks (the mid-intervention point). The information provision component was the second most used part of the app, with 55% (335/609) of the users accessing the toolkit at least once. The lowest level of engagement was observed in the social support component, with 29.1% (177/609) of the users accessing it at least once. The users of the social support feature made between 0 and 37 posts (mean 0.5, SD 2.7), 0 to 96 likes (mean 0.5, SD 4.6), and 0 to 17 comments (mean 0.4, SD 1.7).

**Figure 1:**
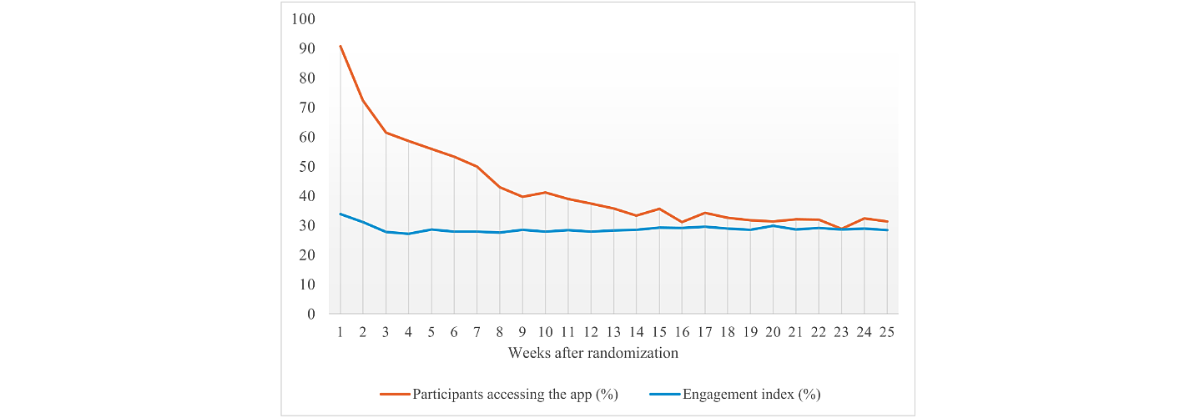
Average weekly engagement index (EI) over the use period for the participants defined as users (n=609). The line for the participants accessing the app (%), as indicated in the legend, shows the proportion of participants who accessed the app out of the 609 users. The EI (%) comprises 4 subindexes: click depth (the average number of app pages viewed per day), loyalty (the total number of days a user engaged with the app), recency (the average number of days between app use: reversed so higher score indicates greater engagement), and diversity (the number of different app features used per day). The subindexes were normalized by rescaling values between 0 and 100 to assign equal weight to each of the subindexes (each element was equally important in contributing to the measurement of engagement). The EI was then calculated as the arithmetic mean of the 4 subindexes (click depth, loyalty, recency, and diversity).

The average EI over the 25 weeks was 28% (SD 7%) out of 100%. Regarding the patterns of engagement, the level of engagement (EI) varied throughout the 25 weeks (Figure 1). For those who progressed past the log-in page, the engagement was highest after initial access to the app (552/609, 90.6% of users accessed the app in the first week; average EI in the first week was 34%, SD 12%) and declined during the first 25 weeks of the intervention period (227/609, 37.3% of users accessed the app at 12 weeks and 190/609, 31.2% at 25 weeks) although in a nonlinear fashion. For users with engagement data at both 12 and 25 weeks, the EI was similar at the 2 time points (mean 28%, SD 7%; 217/609, 35.6% and mean 28%, SD 9%; 190/609, 31.2%, respectively).

### Associations Between the Predictors and User Engagement

The estimated associations between the potential predictors and the individual (time spent in the app, proportion of pages visited, clicks in the main parts of the app) and the composite (EI and subindexes) indicators of engagement from the unadjusted regression models are presented in Tables 3, 4, and 5, respectively. Overall, the same pattern of associations was observed for the individual and composite measures of engagement.

Higher education level was associated with higher levels of engagement on 4 of the 7 individual indicators and all 5 of the composite indicators. Higher education level was associated with more days of app use (b=3.14, 95% CI 0.50-5.78; *P*=.02); accessing a higher proportion of pages (b=3.27, 95% CI 0.96-5.59; *P*=.006) and features (b=4.33, 95% CI 1.05-7.61; *P*=.01); and more clicks in the modules (b=18.59, 95% CI 5.33-31.86; *P*=.006), as well as higher EI (b=2.61, 95% CI 1.46-3.77; *P*<.001); click depth (b=0.77, 95% CI 0.36-1.19; *P*<.001); loyalty (b=2.48, 95% CI 1.14-3.83; *P*<.001); recency (b=4.64, 95% CI 2.00-7.27; *P*=.001); and diversity index (b=2.56, 95% CI 0.89-4.24; *P*=.003). The parent coping score was positively associated with engagement on 1 composite indicator (ie, loyalty score: b=1.25, 95% CI 0.43-2.08; *P*=.003).

In contrast, higher working hours were associated with lower levels of engagement on 4 of the individual indicators and 1 of the composite indicators. Number of working hours was inversely associated with total time spent in the app (b=−0.24, 95% CI −0.43 to −0.05; *P*=.01); proportion of pages visited (b=−0.08, 95% CI −0.15 to −0.01; *P*=.02); and clicks in the modules (b=−0.40, 95% CI −0.79 to −0.01; *P*=.046) and toolkit (b=−0.27, 95% CI −0.48 to −0.06; *P*=.01), as well as click-depth index (b=−0.01, 95% CI −0.03 to −0.00; *P*=.046). Parents living together were associated with lower levels of engagement on 5 of the individual indicators. Parents living together with a partner spent less time (b=−13.58, 95% CI −24.39 to −2.77; *P*=.01) and days using the app (b=−5.07, 95% CI −9.531 to −0.6; *P*=.03); accessed a lower proportion of pages (b=−4.30, 95% CI −8.22 to −0.38; *P*=.03); and, on average, clicked less times in the modules (b=−31.37, 95% CI −53.79 to −8.95; *P*=.006) and toolkit (b=−12.72, 95% CI 24.82-0.63; *P*=.04). Having siblings in the family was associated with lower engagement on 5 of the individual indicators and 4 of the composite indicators. Having siblings in the family was inversely associated with total time spent in the app (b=−7.11, 95% CI −13.40 to -0.82; *P*=.03); number of days of app use (b=−3.04, 95% CI −5.59 to −0.49; *P*=.02); and clicks in the toolkit (b=−7.75, 95% CI −14.82 to −0.68; *P*=.03), as well as lower EI (b=−1.56, 95% CI −2.65 to −0.48; *P*=.005); click depth (b=−0.43, 95% CI −0.83 to −0.04; *P*=.03); loyalty (b=−2.4, 95% CI −3.68 to −1.12; *P*<.001); and recency index (b=−2.77, 95% CI −5.17 to −0.36; *P*=.02). Living outside a metropolitan area was associated with lower engagement on 1 of the composite indicators (ie, click-depth index: b=−0.47; 95% CI −0.88 to −0.05; *P*=.03).

No associations were observed between any of the predictor variables and clicks in the community chat forum. Parental characteristics (age and birth country), information seeking and concern, and area-level socioeconomic status were not associated with any of the considered indicators of engagement.

The sensitivity analyses yielded similar results, with a few exceptions. The associations between education level and total time spent in the app were significant after adjustment for app downtime (β=6.61, 95% CI 0.21-13.00; *P*=.04) and days in lockdown (β=6.75, 95% CI 0.35-13.16; *P*=.04). In contrast, associations between work status and click-depth index (β=−0.01, 95% CI −0.02 to −0.00; *P*=.05); between siblings in the family and the proportion of features visited (β=−2.90, 95% CI −6.00 to 0.24; *P*=.07); and between remoteness (major city vs regional or remote area) and click-depth index (β=−0.32, 95% CI −0.73 to 0.10; *P*=.14) were attenuated after adjustment for app downtime and days in lockdown.

**Table 3:** Associations between family demographics and parental characteristics with the individual engagement indicators for overall use (n=609)^a^.

Predictor variables	Total time spent in the app (min)	App use (d)	Proportion of pages visited (%)	Proportion of features visited (%)
	β^b^ (95% CI)	β (95% CI)	β (95% CI)	β (95% CI)
Parent age (y)	0.45 (−0.19 to 1.09)	0.10 (−0.16 to 0.37)	0.13 (−0.10 to 0.36)	0.12 (−0.21 to 0.45)
Australian-born	−0.91 (−7.71 to 5.90)	0.22 (−2.58 to 3.03)	−0.14 (−2.61 to 2.32)	0.64 (−2.84 to 4.13)
University degree^c^	6.30 (−0.11 to 12.71)	*3.14 (0.50 to 5.78)* ^d^	*3.27 (0.96 to 5.59)*	*4.33 (1.05 to 7.61)*
Work status (h/wk)	−*0.24 (−0.43 to −0.05)*	−*0.04 (−0.12 to 0.04)*	−*0.08 (−0.15 to −0.01)*	−0.08 (−0.18 to 0.02)
Parents living together	−*13.58 (−24.39 to −2.77)*	−*5.07 (−9.53 to −0.61)*	−*4.30 (−8.22 to −0.38)*	−4.51 (−10.06 to 1.05)
Siblings in the family	−*7.11 (−13.4 to −0.82)*	−*3.04 (−5.59 to −0.49)*	−*2.66 (−4.92 to −0.40)*	−*3.22 (−6.38 to −0.07)*
Living in a regional area^e^	−1.62 (−7.97 to 4.72)	−0.95 (−3.57 to 1.66)	−1.00 (−3.29 to 1.30)	−1.49 (−4.75 to 1.76)
SEIFA^f^ score	0.01 (−0.04 to 0.05)	0.01 (−0.01 to 0.02)	0.01 (−0.01 to 0.02)	0.01 (−0.01 to 0.03)
Parent coping	1.70 (−2.23 to 5.62)	0.99 (−0.62 to 2.61)	0.32 (−1.11 to 1.74)	0.56 (−1.45 to 2.58)
Concern with child MB^g^	−0.53 (−1.61 to 0.55)	−0.20 (−0.65 to 0.24)	−0.20 (−0.59 to 0.19)	−0.28 (−0.01 to 0.30)
Information seeking	0.33 (−0.31 to 0.98)	−0.04 (−0.31 to 0.23)	−0.03 (−0.21 to 0.26)	−0.06 (−0.39 to 0.27)

^a^Due to missing data, n varied slightly for some models, ranging from 1 to 62 missing observations.

^b^β: unstandardized regression coefficient.

^c^University degree or higher education versus no university degree.

^d^Italics indicate statistical significance.

^e^Major cities versus inner or outer regional and remote Australia.

^f^SEIFA: Socio-Economic Indexes for Areas.

^g^MB: movement behaviors.

**Table 4:** Associations between family demographics and parental characteristics with the individual indicators for engagement in the main parts of the app (ie, clicks in the modules, toolkit, and community chat forum; n=609)^a^.

Predictor variables	Learning module clicks (n)	Toolkit clicks (n)	Community chat forum clicks (n)
	β^b^ (95% CI)	β (95% CI)	β (95% CI)
Parent age (y)	0.71 (−0.62 to 2.04)	0.64 (−0.08 to 1.35)	0.00 (−0.08 to 0.09)
Australian-born	−3.22 (−17.4 to 10.9)	−0.40 (−8.00 to 7.21)	−0.01 (−0.92 to 0.90)
University degree^c^	*18.59* ^d^ *(5.32 to 31.86)*	5.34 (−1.83 to 12.51)	0.74 (−0.12 to 1.60)
Work status (h/wk)	−*0.40 (−0.79 to −0.01)*	−*0.27 (−0.48 to −0.06)*	−0.02 (−0.04 to 0.01)
Parents living together	−*31.37 (−53.79 to −9.00)*	−*12.72 (−24.82 to −0.63)*	−0.50 (−1.95 to 0.95)
Siblings in the family	−10.92 (−23.76 to 1.92)	−*7.75 (−14.82 to −0.68)*	−0.58 (−1.41 to 0.24)
Living in a regional area^e^	−6.37 (−19.55 to 6.81)	−3.51 (−10.60 to 3.58)	−0.13 (−0.98 to 0.72)
SEIFA^f^ score	0.03 (−0.05 to 0.12)	0.01 (−0.04 to 0.05)	0.00 (−0.00 to 0.01)
Parent coping	5.89 (−2.26 to 14.03)	−2 (−6.39 to 2.39)	−0.10 (−0.63 to 0.42)
Concern with child MB^g^	−1.07 (−3.31 to 1.17)	−0.63 (−1.84 to 0.58)	0.01 (−0.14 to 0.15)
Information seeking	0.46 (−0.88 to 1.80)	0.04 (−0.65 to 0.73)	0.01 (−0.07 to 0.10)

^a^Due to missing data, n varied slightly for some models, ranging from 1 to 62 missing observations.

^b^β: unstandardized regression coefficient.

^c^University degree or higher education versus no university degree.

^d^Italics indicate statistical significance.

^e^Major cities versus inner or outer regional and remote Australia.

^f^SEIFA: Socioeconomic Indexes for Areas.

^g^MB: movement behaviors.

**Table 5:** Associations of family demographics and parental characteristics with the composite indicators of engagement (n=609)^a^.

Predictor variables	Total score	Click depth	Loyalty	Recency	Diversity
	β^b^ (95% CI)	β (95% CI)	β (95% CI)	β (95% CI)	β (95% CI)
Parent age (y)	0.07 (−0.05 to 0.18)	0.02 (−0.02 to 0.06)	0.04 (−0.10 to 0.17)	0.11 (−0.16 to 0.38)	0.10 (−0.07 to 0.27)
Australian-born	−0.99 (−2.21 to 0.23)	−0.32 (−0.77 to 0.12)	−0.99 (−2.43 to 0.44)	−1.87 (−4.62 to 0.88)	−0.78 (−2.56 to 1)
University degree^c^	*2.61 (1.46 to 3.77)* ^d^	*0.77 (0.36 to 1.19)*	*2.48 (1.14 to 3.83)*	*4.64 (2 to 7.27)*	*2.56 (0.89-4.24)*
Work status (h/wk)	−0.02 (−0.06 to 0.01)	*-0.01 (-0.03 to -0.00)*	−0.03 (−0.07 to 0.02)	−0.04 (−0.12 to 0.04)	−0.02 (−0.07 to 0.03)
Parents living together	−0.29 (−2.27 to 1.68)	−0.44 (−1.15 to 0.27)	−2.15 (−4.43 to 0.15)	1.55 (−2.94 to 6.04)	−0.14 (−2.99 to 2.71)
Siblings in the family	−*1.56 (−2.65 to −0.48)*	−*0.43 (−0.83 to −0.04)*	−*2.40 (−3.68 to −1.12)*	−*2.77 (−5.17 to −0.36)*	−0.65 (−2.24 to 0.93)
Living in a regional area^e^	−0.20 (−1.36 to 0.95)	−*0.47 (−0.88 to −0.05)*	0.07 (−1.27 to 1.41)	−0.14 (−2.77 to 2.48)	−0.27 (−1.93 to 1.40)
SEIFA^f^ score	−0.00 (−0.01 to 0.01)	0.00 (−0.00 to 0.00)	−0.00 (−0.01 to 0.01)	0.00 (−0.01 to 0.02)	−0.00 (−0.02 to 0.01)
Parent coping	0.56 (−0.15 to 1.27)	0.13 (−0.13 to 0.39)	*1.25 (0.43-2.08)*	0.62 (−1.01 to 2.24)	0.24 (−0.79 to 1.27)
Concern with child MB^g^	0.03 (−0.17 to 0.23)	0.00 (−0.07 to 0.08)	−0.07 (−0.30 to 0.16)	−0.06 (−0.50 to 0.39)	0.25 (−0.04 to 0.53)
Information seeking	0.04 (−0.08 to 0.16)	0.03 (−0.02 to 0.07)	−0.04 (−0.17 to 0.10)	0.07 (−0.20 to 0.34)	0.09 (−0.08 to 0.26)

^a^Due to missing data, n varied slightly for some models, ranging from 1 to 62 missing observations.

^b^β: unstandardized regression coefficient.

^c^University degree or higher education versus no university degree.

^d^Italics indicates statistical significance.

^e^Major cities versus inner or outer regional and remote Australia.

^f^SEIFA: Socioeconomic Indexes for Areas.

^g^MB: movement behaviors.

## Discussion

### Principal Findings

To the best of our knowledge, this is the first mHealth intervention study targeting parents of young children to examine user engagement and predictors of engagement beyond simple demographics. In addition to including family demographics, our study also considered parental cognitions and behaviors relevant to the intervention, which are crucial for interventions targeting parents as agents of change for child behavior. Thus, our findings contribute to an increased understanding of which factors influence parental engagement in digital interventions and who may need more active strategies to support their engagement in digital interventions. The use of the Let’s Grow app was initially high, with declining levels; however, among those who continued to use the app across the 25-week period, the level of engagement remained consistent across this timeframe. We identified several demographic predictors of engagement. More work hours, parents living together, having siblings in the family, and living in a regional or remote area were associated with lower engagement, while having a higher education level was associated with higher engagement. Of the parental characteristics examined, higher parent coping was positively associated with engagement, while parent concern and information seeking in relation to child movement did not appear to impact engagement.

### Comparison With Previous Findings

User engagement in digital interventions has gained more interest in recent years, and the link between intervention uptake and efficacy has been established [25]. However, the conceptualization and reporting of engagement in digital interventions targeting parents and other populations (eg, Saleem et al [26]) remain scarce, and there are no universal criteria for defining *good* engagement. Nevertheless, engagement has been the focus of commercial apps and website companies for a long time. A report on engagement in all available apps in the Apple and Google Play stores suggests that 25% of all downloaded apps are used only once in the first 6 months [27], and an engagement rate between 1% and 5% has been described as acceptable app engagement [28,29]. In this context, initial engagement in our study can be described as high, which is also similar to what has been observed in previous studies investigating engagement in digital health interventions (eg, [29,30]). For instance, Røed et al [13] reported that 13.5% (20/148) of the enrollees in their study did not enter the website at all over the 6-month intervention period, similar to 10.7% (73/682) in our study. Moreover, our results showed that, on average, users accessed the app for more than 18 days (equivalent to approximately once a week), which is similar to the findings of the MINISTOP trial [31] in which parents of children aged 2 to 3 years used the intervention app to register child health behaviors on average once per week during the 6-month intervention period. Furthermore, overall engagement with our app, as assessed by the mean composite EI (4 subindexes), was moderate and almost identical to that reported in 2 other studies that investigated engagement in the Growing Healthy app among expectant parents and parents with an infant [11] and the Milk Man app among fathers with an infant [14]. Both the Growing Healthy and the Milk Man studies also used an adapted version of the Web Analytics Demystified visitor index (5 subindexes) used in this study and reported a composite mean EI of 30% (SD 12%; range 2%-58%) and 30% (SD 20%; range 1%-80%), respectively. Similarly, a study involving parents (n=42) of children aged 2 to 5 years reported a slightly higher index score (median 56, SD 21; range 4-81; scale 0-100) with an mHealth parent-training program (ezPARENT tablet app); however, they used a different index based on only 3 engagement measures (ie, the completion of app modules, number of visits, and time between visits) [12].

Although efforts to conceptualize engagement with digital health interventions have been made [32-34], to date, there is no standard measurement approach for engagement in mHealth interventions. This makes it difficult to synthesize the evidence and compare engagement across studies. Considering the differences in intervention design and thus purposive tailoring and calculation of these EIs, direct comparison between studies is impractical. Nevertheless, quantifying engagement should still be a priority, and for that purpose, it can be argued that a composite EI can serve as a study-specific indicator of user engagement and may be a valuable tool for statistical analysis purposes (eg, investigating intervention effects). However, careful deliberation is required to include the most relevant indicators for that intervention. Moreover, individual indicators of engagement, such as total time spent using the app and frequency of engagement, may be better for direct comparison between apps, as they are indicators that all apps can extract and are likely more feasible for researchers to use. A further complication in assessing engagement is that some apps, such as the Let’s Grow app, act as tools to promote offline engagement in behavior change, which is not measurable through app use metrics. For example, most of the activities in the behavior change component of the app were designed to prompt parents to engage their children in active play opportunities offline. Engagement in these offline components of the intervention, which form a key aspect of intervention dose and are likely to impact intervention efficacy, is not captured in app engagement statistics. Thus, future studies should aim to develop and assess measures beyond objective engagement metrics to enable a complete assessment of engagement in mHealth interventions that promote offline activities.

Another important aspect of participant engagement is use over time, and retaining user’s engagement in digital health interventions has been reported to be challenging [35,36]. Our pattern analyses indicate that use was not linear. Rather, we found an initial decline during the first few weeks, followed by steady levels of engagement among the subgroup who were still using the intervention at 12 and 25 weeks. In contrast, Taki et al [11] showed a downward trend in engagement subindexes score from 12 to 25 weeks, which aligns with studies in other populations in which continual linear decline in use over time has been reported [37,38]. One possible reason for the difference between our findings and the consistent declines reported for the Growing Healthy app [11] and other mHealth interventions (eg, the HealthyMoms app [39]) may lie in a key design difference. To illustrate, in the Growing Healthy app, all content was freely available whenever the user wished to access it [40], and new intervention content was delivered regularly (ie, biweekly) in the HealthyMoms app regardless of participant use [39]. In contrast, Let’s Grow participants had to work through modules to unlock the next one. This may have encouraged more regular, ongoing use among those highly engaged parents with continued use across the study and could be viewed as a form of gamification (users were encouraged to keep coming back to unlock new content), which has been described as somewhat successful in achieving maintained parental engagement in child health promotion apps [36]. However, it is also possible that not having all content freely available or the need to undertake behavior change activities (rather than only viewing content) in Let’s Grow may have been a barrier to engagement for some participants. A better understanding of engagement with these and other app designs, such as individually tailored apps (eg, using a just-in-time adaptive intervention design), is needed. Moreover, formal testing is required to understand what format best optimizes engagement, as well as to develop implementation strategies that support and sustain user engagement.

Furthermore, a better understanding of the participant characteristics that impact engagement in parental interventions can assist in clarifying how to design interventions to maximize behavior change. Nevertheless, a few previous studies [11-14] have reported predictors of engagement in parental mHealth interventions, and they have mostly focused on parent and family demographic characteristics. In our study, we expanded this to include 3 measures of parental characteristics, including parent coping in general as well as concern and information seeking specific to the targeted topic (child movement behaviors). We identified several predictors related to time and socioeconomic pressures (education level, work hours, remoteness, and family structure), as well as parent coping in general (not specific to the targeted intervention topic). In more detail, socioeconomic pressures, such as lower education level, working more hours, and living in a remote or outer regional area, as well as family structure (living with a partner or spouse and siblings in the family), were associated with *lower* engagement. This is consistent with previous studies investigating predictors of engagement in parental mHealth interventions, which have shown lower levels of engagement among parents with lower education levels [13] and those with more than 1 child [11,13]. Indeed, more work hours and having more than 1 child could be seen as indicators of less available time, which has previously been described as a barrier to app engagement [37]. Moreover, poorer parental coping in general was identified as a predictor of *lower* engagement. Similar to the lack of available time, this may reflect a lack of parent capacity to take on new learning when they are struggling to cope with their daily commitments. In contrast, perceived need, motivation, and utility have been described as factors that promote engagement in health apps for adults [35,41,42]. Similarly, a mixed methods assessment in a subsample from the Growing Healthy study showed that participants defined as having low or moderate engagement felt they had sufficient knowledge and that the app did not provide further information [43]. In our study, parents who did not engage with the app (defined as nonusers) reported lower coping scores and less concern about movement behaviors compared to those defined as users. Unexpectedly and notably, parental concern about child movement behaviors and parent information seeking about topics related to the intervention content were also not associated with engagement in our study. This indicates that being concerned and coping with life may predict actually starting the intervention (ie, initial engagement), but beyond enrolling in the study, it seems that other factors drive how much parents engage. Altogether, this suggests that time constraints and other pressures may outweigh the content relevance in determining actual engagement with the intervention, and parents under time or other pressures might need more support to initiate and sustain engagement.

### Strengths and Limitations

This study has several strengths, including the objective and accurate assessment of the outcome (ie, participant engagement) and the relatively large sample size. In addition, the use of several engagement metrics provides a more comprehensive understanding of how users interact with mHealth interventions [44-46], and greater insights into engagement can be achieved by aggregating data across different domains (ie, frequency, intensity, time, and type) [46]. Thus, another strength of the study is the reporting of both the individual measures of engagement (representing engagement in 3 key strategies for promoting health behaviors: behavior change, information provision, and social support) and the overall engagement (EI). The study also has some limitations to acknowledge. First, we used mid-intervention data; thus, our results do not capture engagement over the entire intervention period (ie, 12 months). However, the actual minimum time to complete the intervention program was 22 weeks, which is a similar intervention duration compared to previous studies (eg, Røed et al [13]). Second, the data on the potential predictors were self-reported, and some measures, for example, parental concern, were subjective; thus, results may be subject to reporting biases (ie, social desirability). However, we observed a wide variation in the sample in terms of outcomes and predictor variables. Similar to many previous studies (eg, Røed et al [13]), another limitation of the study is that most participants (98.2%) were mothers. Although most participants lived in major cities (71.8%), participants from inner regional (19.2%) and outer regional or remote Australia (8.9%) were also represented, as well as representation across different socioeconomic areas. In addition, a larger proportion of the participants in the study had a university degree or a higher level of education compared to women in the same age group (25-44 years) in the general population (72% vs 50%) [47]. Nevertheless, our study sample was similar to the general population in terms of birth country (77% vs 71% Australian-born) [47] and work status among women in this age group (65% vs 58%) [47]. Third, we did not measure enactment (ie, the participants’ “real-world” use of intervention skills); thus, we do not have information on intervention engagement beyond use within the app, which was actually designed to encourage parents to enact changes offline. Future studies should aim to capture offline engagement as well, potentially through check-in questions during the intervention period. Fourth, a potential limitation of the study is that we only assessed app engagement data for those who used the app and not for all enrollees (ie, excluding the 73/682, 10.7% of the participants who never used the app), which might have exaggerated engagement. However, the differences in engagement were small, with only slightly lower scores on engagement metrics when all participants were included (eg, mean EI for the whole group was 25.2, SD 10.5 vs 28.0, SD 6.6 for the users only). Nevertheless, nonusers may be qualitatively different from those who have any engagement with the app and are likely to need different strategies to engage them in interventions; our results demonstrate patterns of engagement for those who initiated the use of the app, which is important for informing future app development. Finally, the Let’s Grow app had a nonlinear, dynamic intervention design, where parents could choose the order and timing of their progress, and not all content was freely available at once. This is different from most previously reported studies; thus, some engagement measures may not readily apply. On the same note, engagement scores need to be tailored for individual interventions, and consequently, results are not directly comparable across studies. Acknowledging the tailoring required to specific mHealth interventions, certain metrics, such as the EI used in this study, may provide some level of generalizability across interventions. Therefore, it is ideal for studies to include generalizable and tailored metrics to enable both nuanced assessment of the intervention under investigation and comparison across interventions. However, in cases where such metrics are not available, reporting individual metrics alone should still be a priority.

### Implications and Clinical Relevance

Although the use of mHealth to deliver parental interventions is an established field, and the importance of engagement in interventions has been recognized, engagement in parental mHealth interventions is rarely reported. Thus, our study adds to the scarce literature in several aspects. First, to the best of our knowledge, there is no best practice for assessing engagement in digital interventions, and considering the heterogeneity of intervention designs and delivery, a universal approach may not be feasible, but the inclusion of some generalizable indicators is desirable. Moreover, although the digital format offers a great opportunity to objectively assess engagement, it also represents challenges in deciding which metrics are of importance. In this context, our study reports 2 different approaches to assess participant engagement using both individual metrics and an overall EI and highlights the importance of investigating not only total engagement but also looking at use patterns over time. This information will be useful in investigating potential intervention effects (eg, dose-response relationship), guiding future refinement of the Let’s Grow app and development of similar mHealth interventions, as well as informing the development of guidelines for the assessment and evaluation of user engagement in parental mHealth interventions. Second, reporting of participant engagement and predictors of engagement is important for informing the design and tailoring of future interventions to enhance their use. Moreover, understanding who engaged with the intervention and their patterns of engagement can provide a better understanding of the effect or lack of effect on the targeted outcomes. Third, engagement in digital interventions is often compared to engagement in more traditional face-to-face interventions, which are inherently not comparable due to the differences in intervention delivery and reliance on human interaction. A common assumption is that the digital format (eg, apps) improves reach and makes interventions more accessible for parents, which theoretically should lead to greater engagement. However, as demonstrated in this study, the improved reach may come at the expense of intervention engagement. Although our results show relatively high engagement in comparison to industry reports of app use, they are lower than attendance rates in group-based face-to-face programs, which typically range from 35 to 50% [27,48]. It is interesting to speculate that the lack of reporting of app engagement in mHealth interventions to date could be due to a perception of low engagement relative to what is typically considered *good* engagement through more traditional forms of intervention delivery. It is important for this field of research to report engagement in mHealth interventions so we can accurately gauge any compromise between reach and engagement in this type of intervention delivery. Altogether, our study provides valuable evidence in the field of intervention engagement, which can be of use for the evaluation and design of future parental mHealth interventions.

### Conclusions

This study provides novel data on engagement and predictors of engagement in a parental digital intervention. While engagement data were comparable to other published data, a key difference was the relatively consistent level of engagement noted among individuals showing continued use across the period under investigation, rather than the typically observed continued decline. Our findings show that both family demographics and parental characteristics can influence engagement, and time and socioeconomic pressures might outweigh content relevance when it comes to engagement, although content relevance appears important to initial uptake. Our results highlight the characteristics of parents who may benefit from more active engagement strategies to support their use of digital interventions, specifically those with time and capacity stressors. Finally, our results showed similar outcomes for both the individual and composite measures of engagement, indicating that simpler engagement metrics, which might be more feasible and attainable for researchers to collect, process, and analyze, can be useful in the reporting of engagement in digital interventions. Altogether, our findings will be useful when investigating intervention effects (dose-response relationship) and might be important to consider for future refinement of the Let’s Grow app and the development of similar interventions.

## Appendix

Multimedia Appendix 1 PowerPoint showing screenshots of the Let's Grow app.

Multimedia Appendix 2 Description of the individual subindexes included in the engagement index.

## Abbreviations

EI: engagement index
mHealth: mobile health
REDCap: Research Electronic Data Capture
STROBE: Strengthening the Reporting of Observational Studies in Epidemiology
