# Supplementary material for: Parental and Demographic Predictors of Engagement in an mHealth Intervention: Observational Study From the Let’s Grow Trial
Source: JMIR Mhealth Uhealth. 2025 Jul 15;13:e60478. doi: 10.2196/60478 (PMC12308162; doi:10.2196/60478)

LET'S GROW

IPAN
INSTITUTE FOR PHYSICAL ACTIVITY AND NUTRITION

DEAKIN UNIVERSITY

Overview of the

Let's Grow App

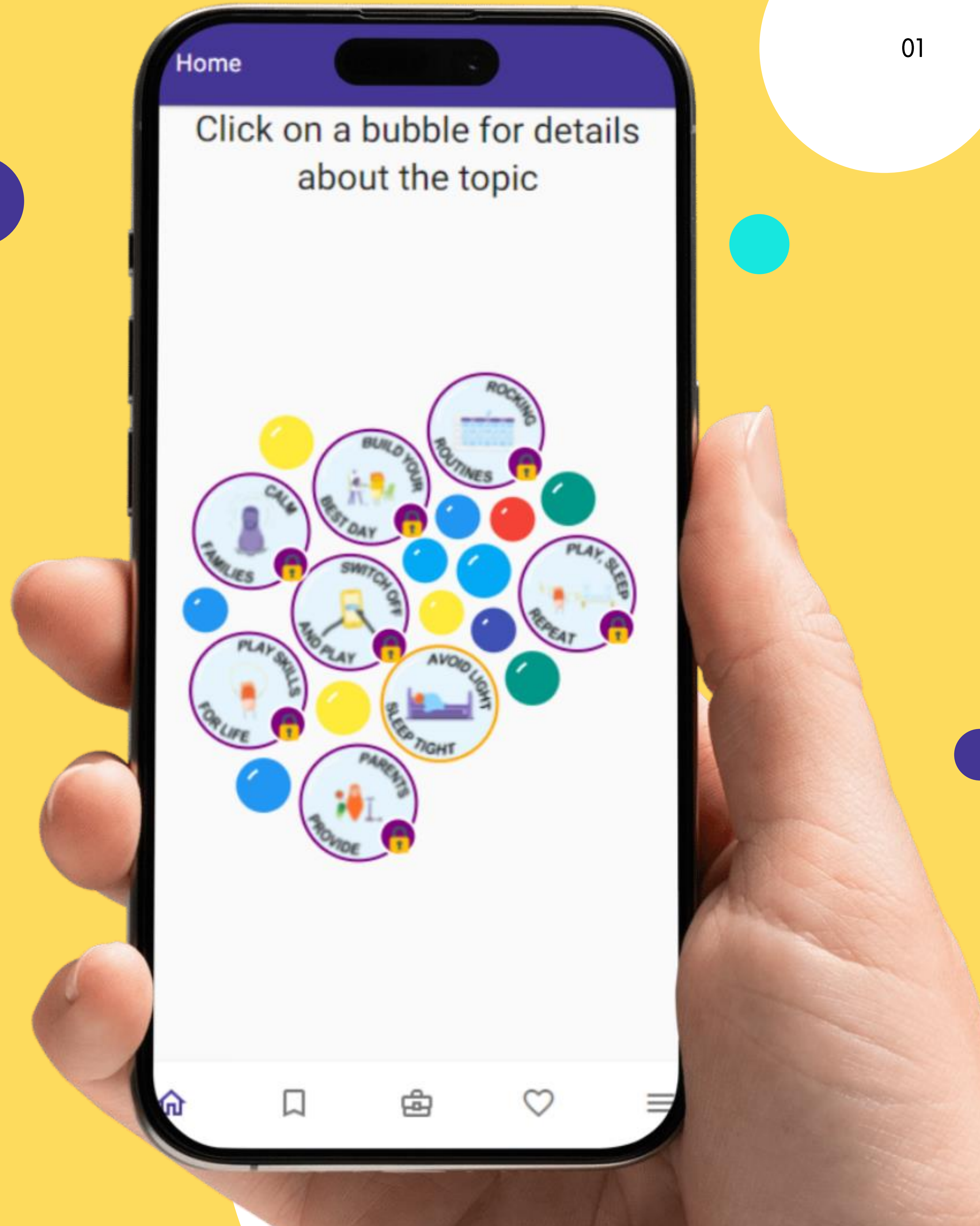

Topic & Activity Overview

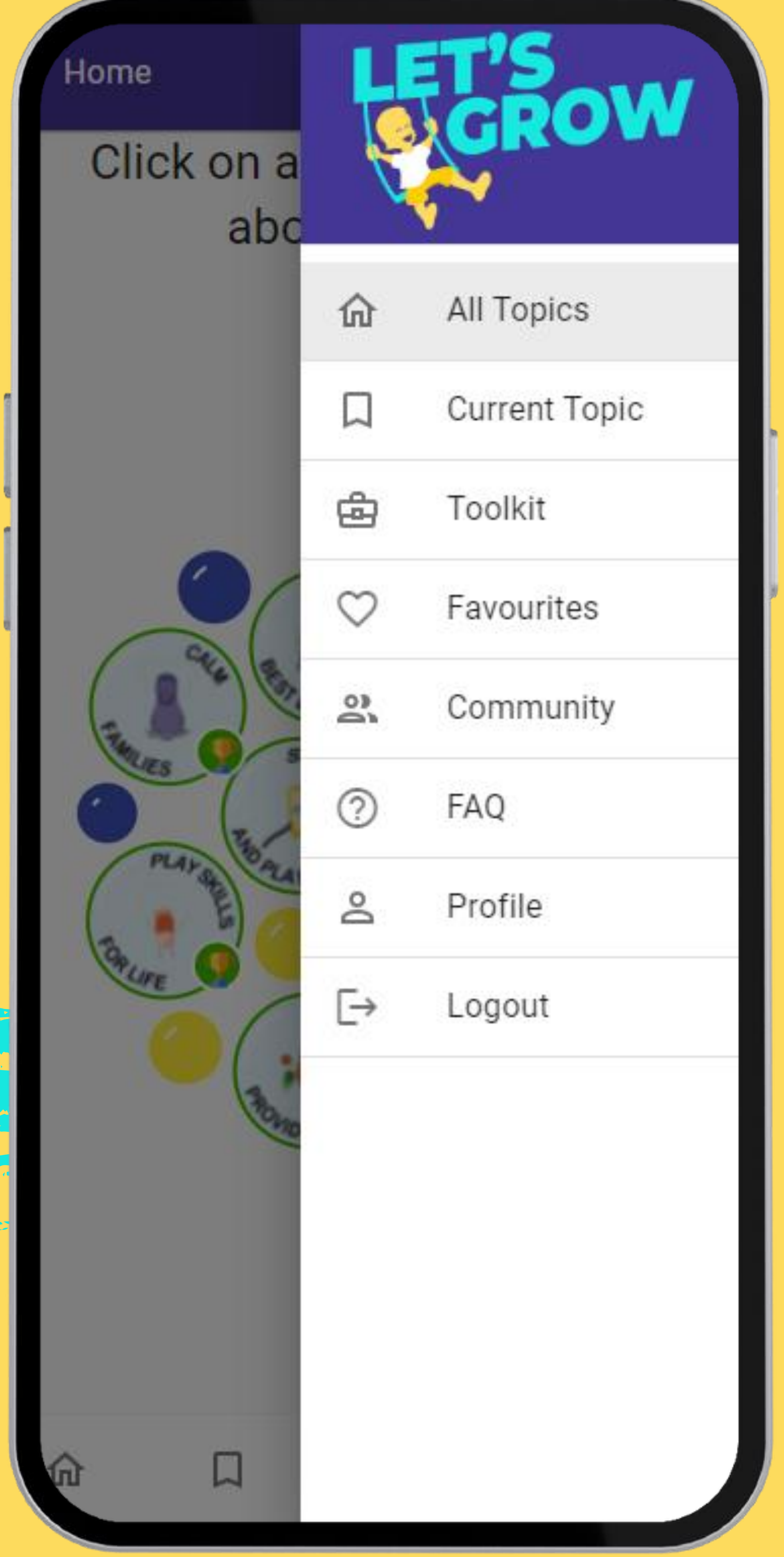

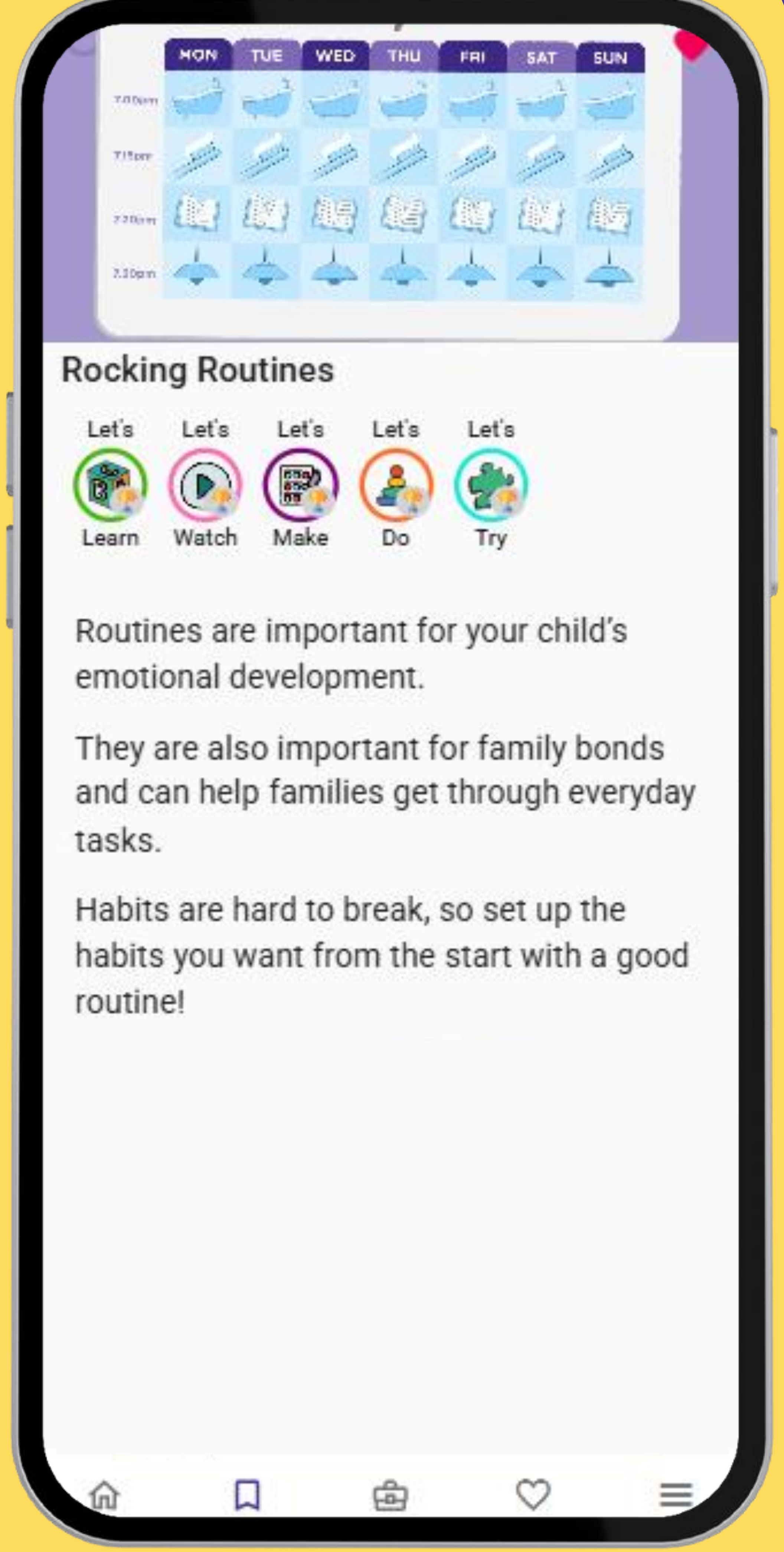

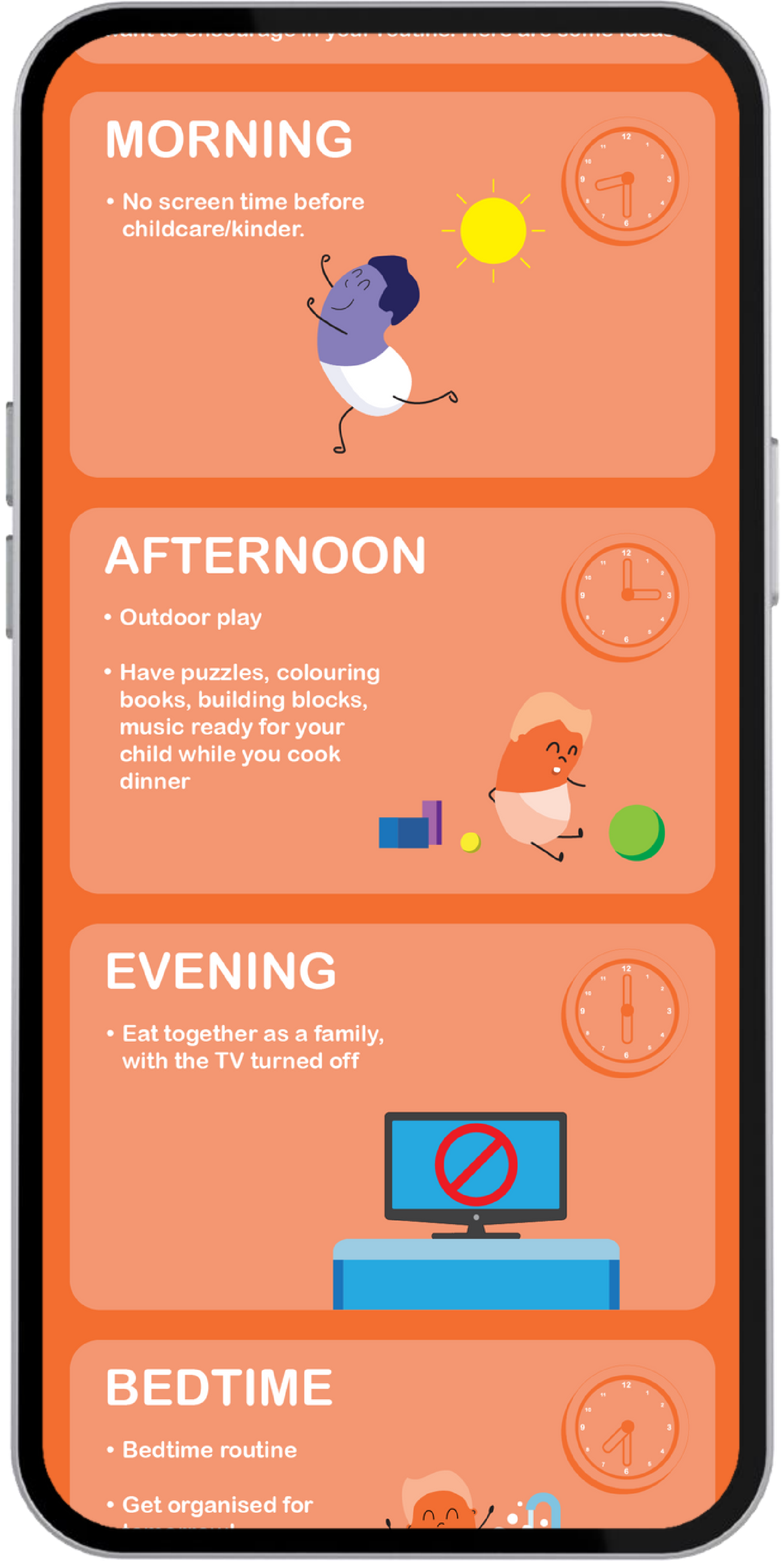

Activity: Infographic

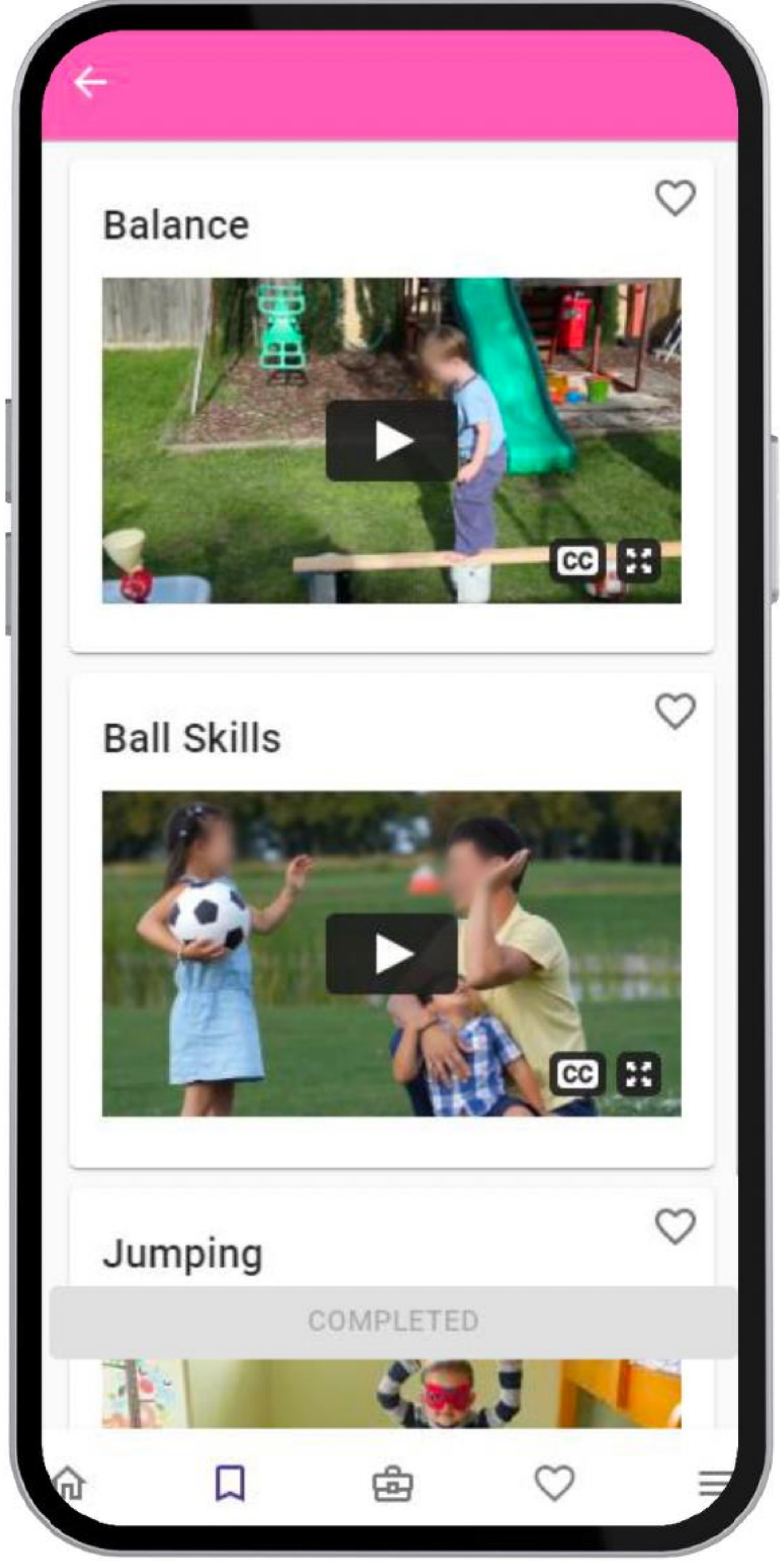

Activity: Video

Activity: Action

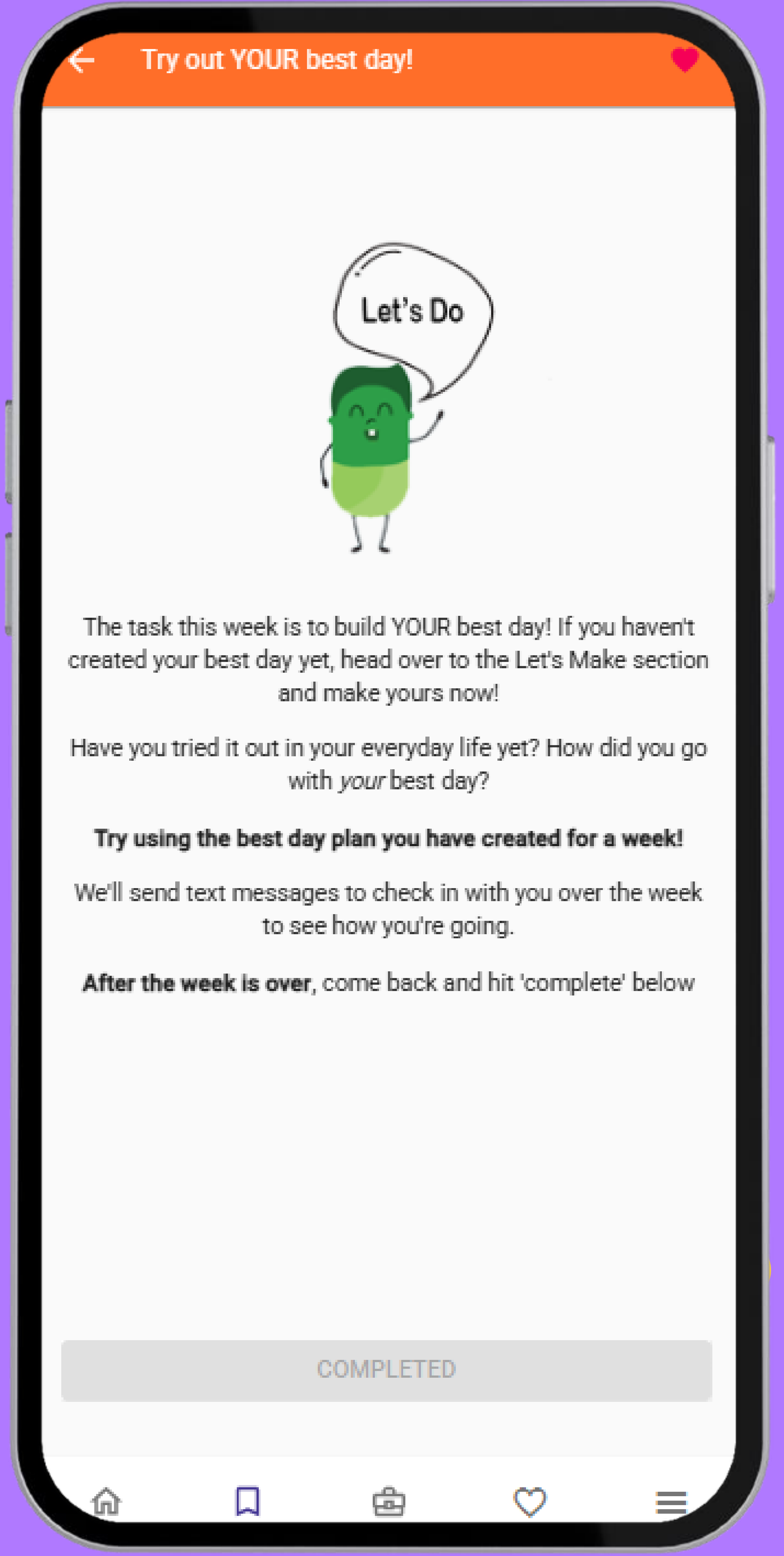

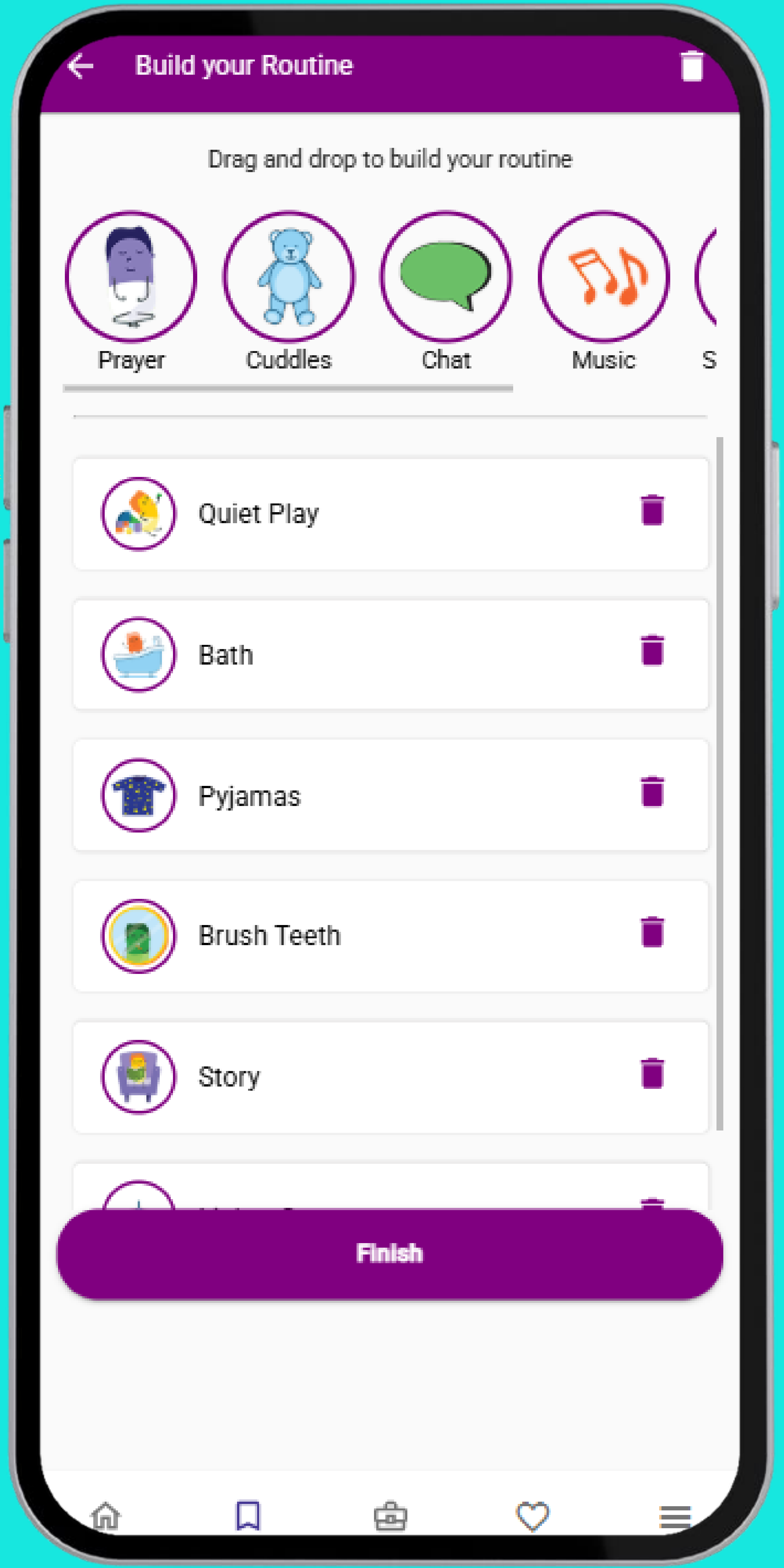

Activity: Routine Builder

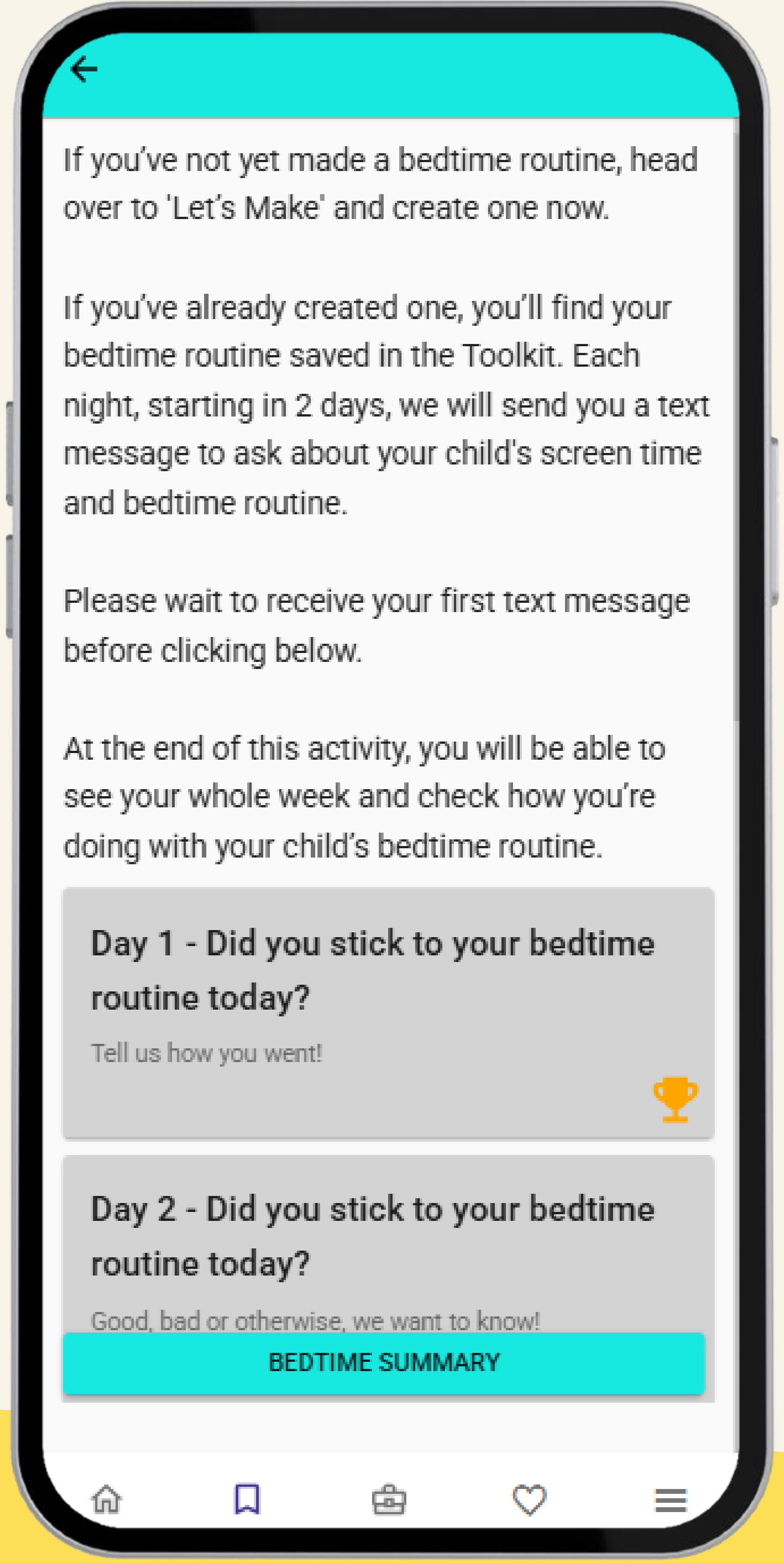

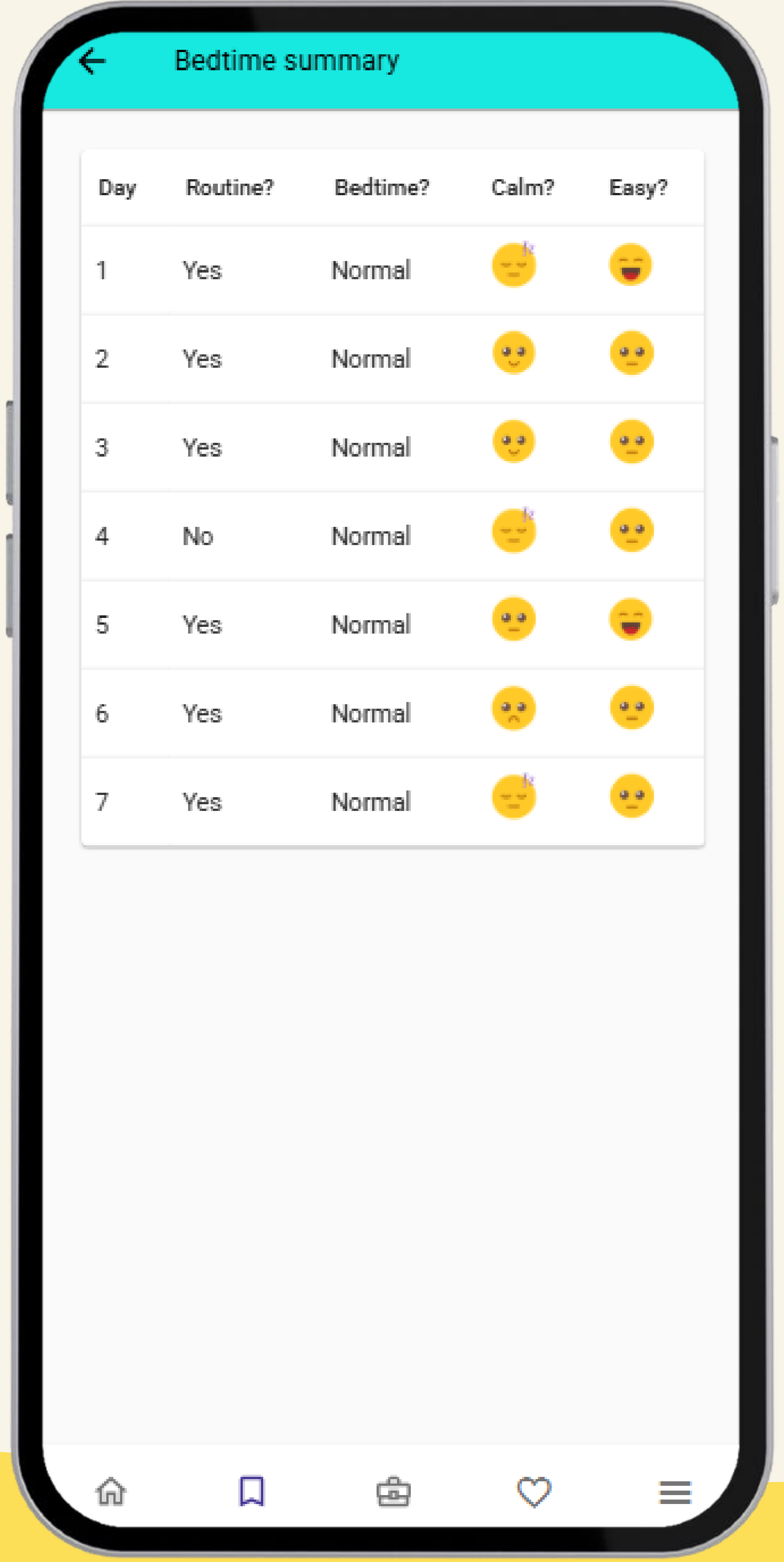

Activity: Self Monitoring

Activity: Goal Setting

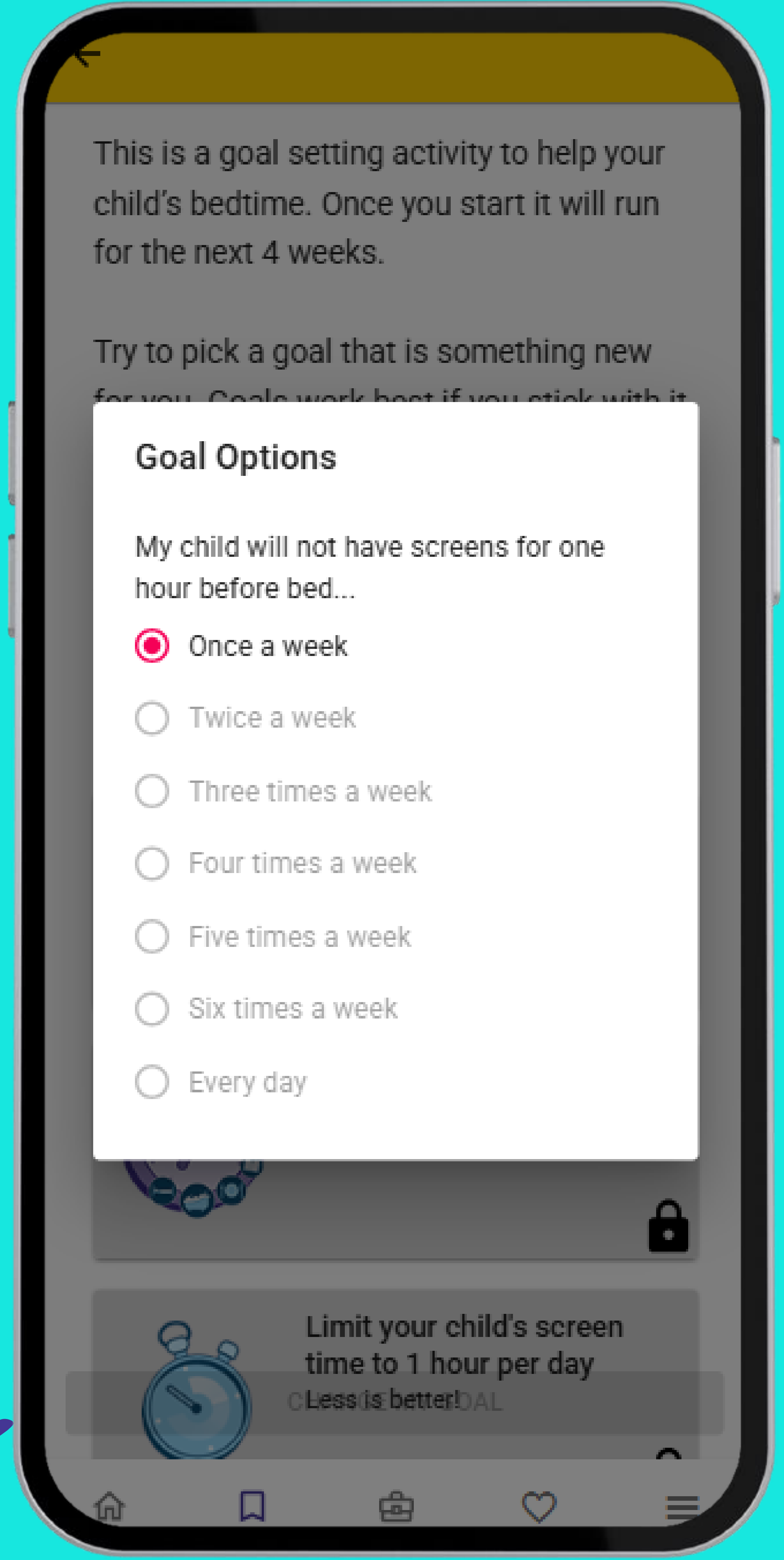

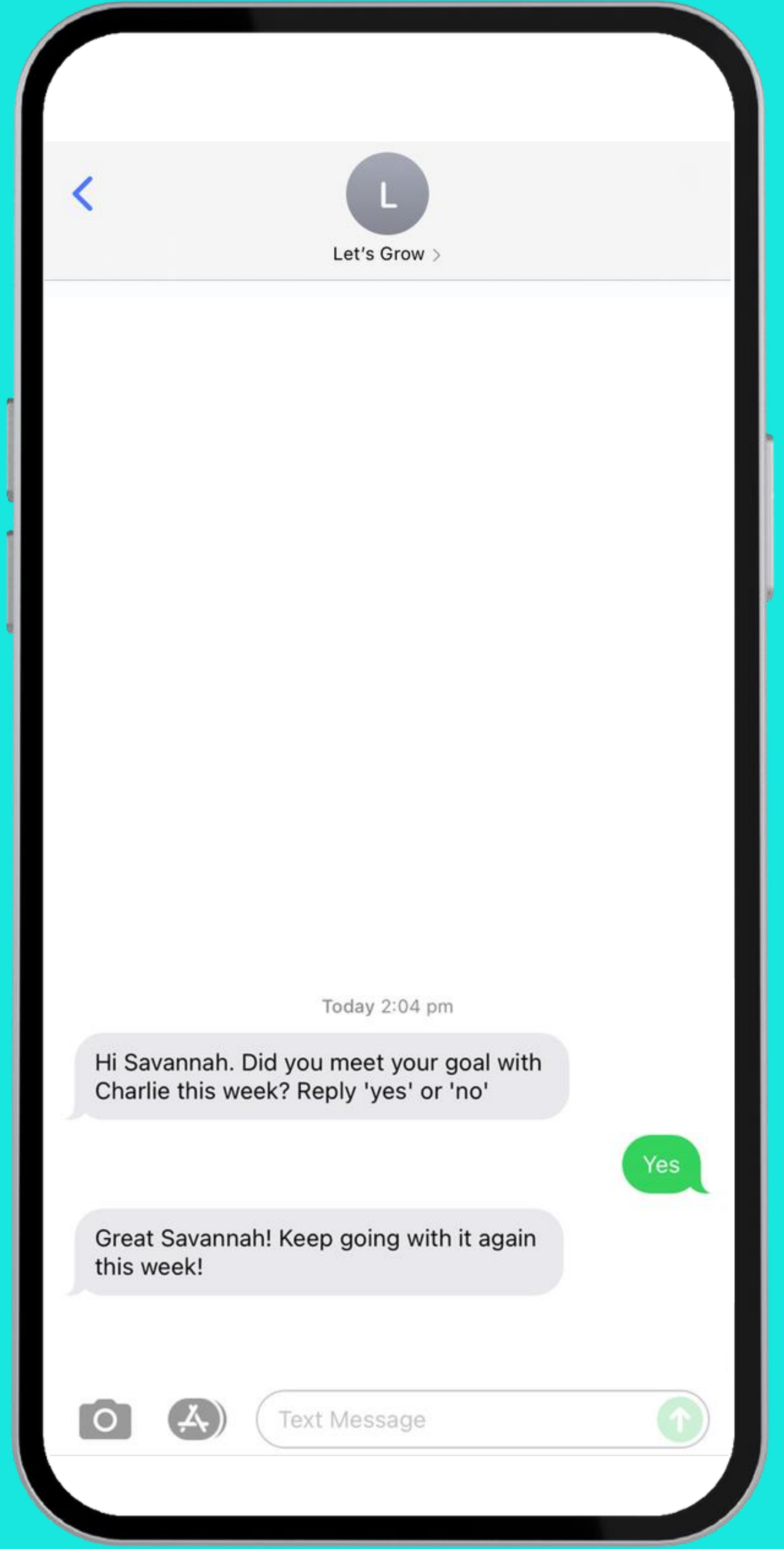

Toolkit

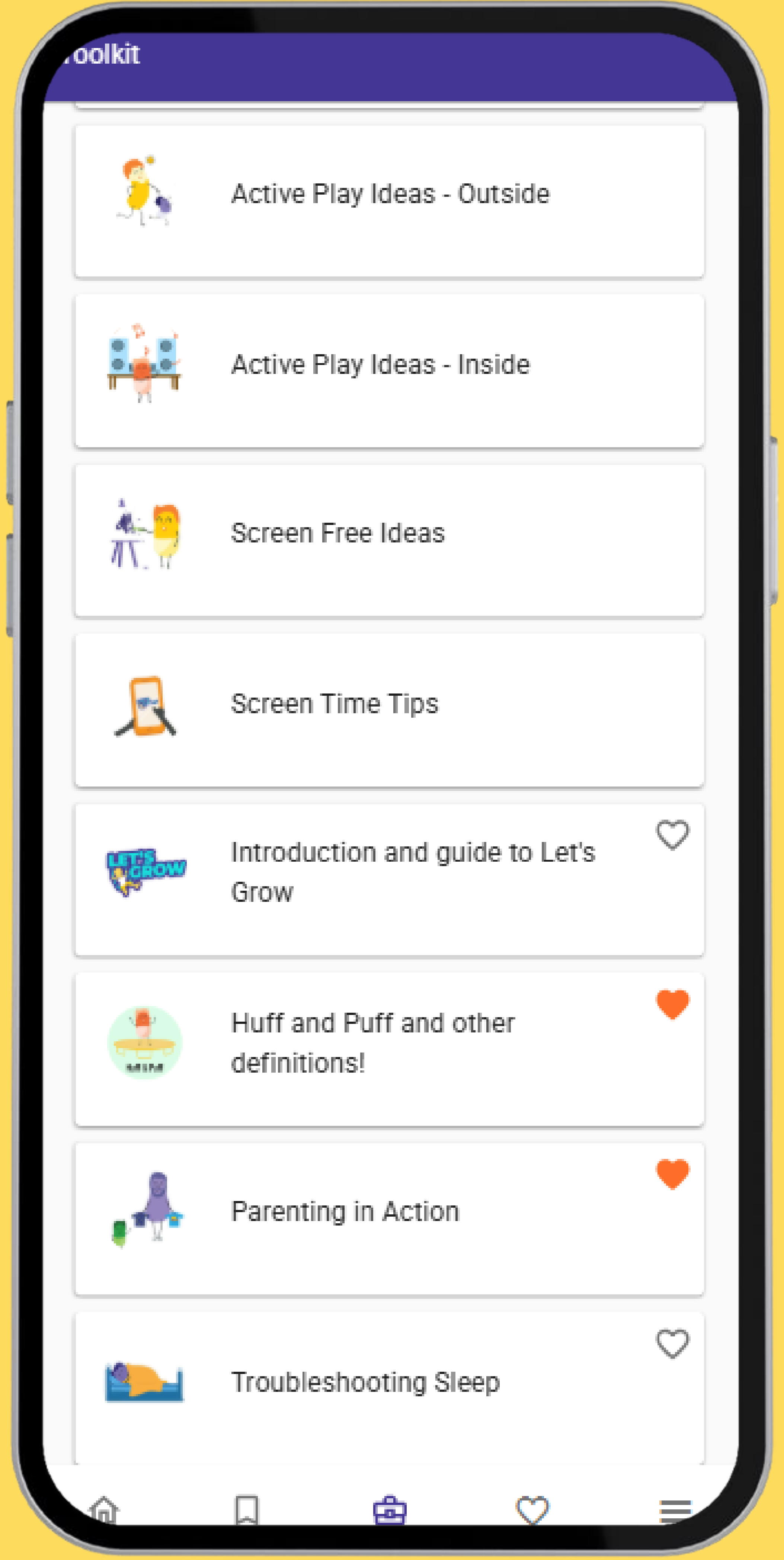

oolkit
Active Play Ideas - Outside
Active Play Ideas - Inside
Screen Free Ideas
Screen Time Tips
Introduction and guide to Let's Grow
Huff and Puff and other definitions!
Parenting in Action
Troubleshooting Sleep

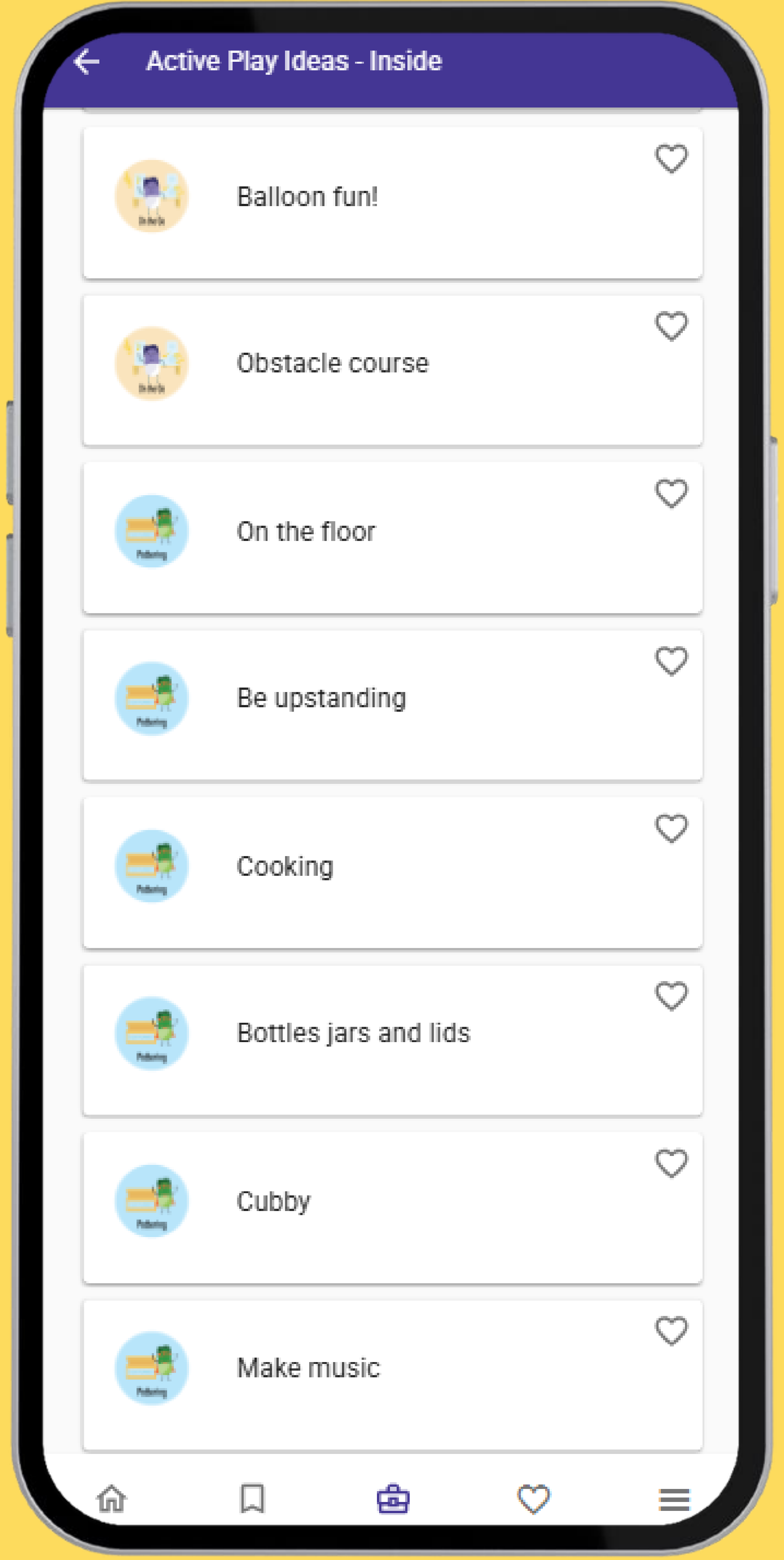

Active Play Ideas - Inside
Balloon fun!
Obstacle course
On the floor
Be upstanding
Cooking
Bottles jars and lids
Cubby
Make music

Community Forum

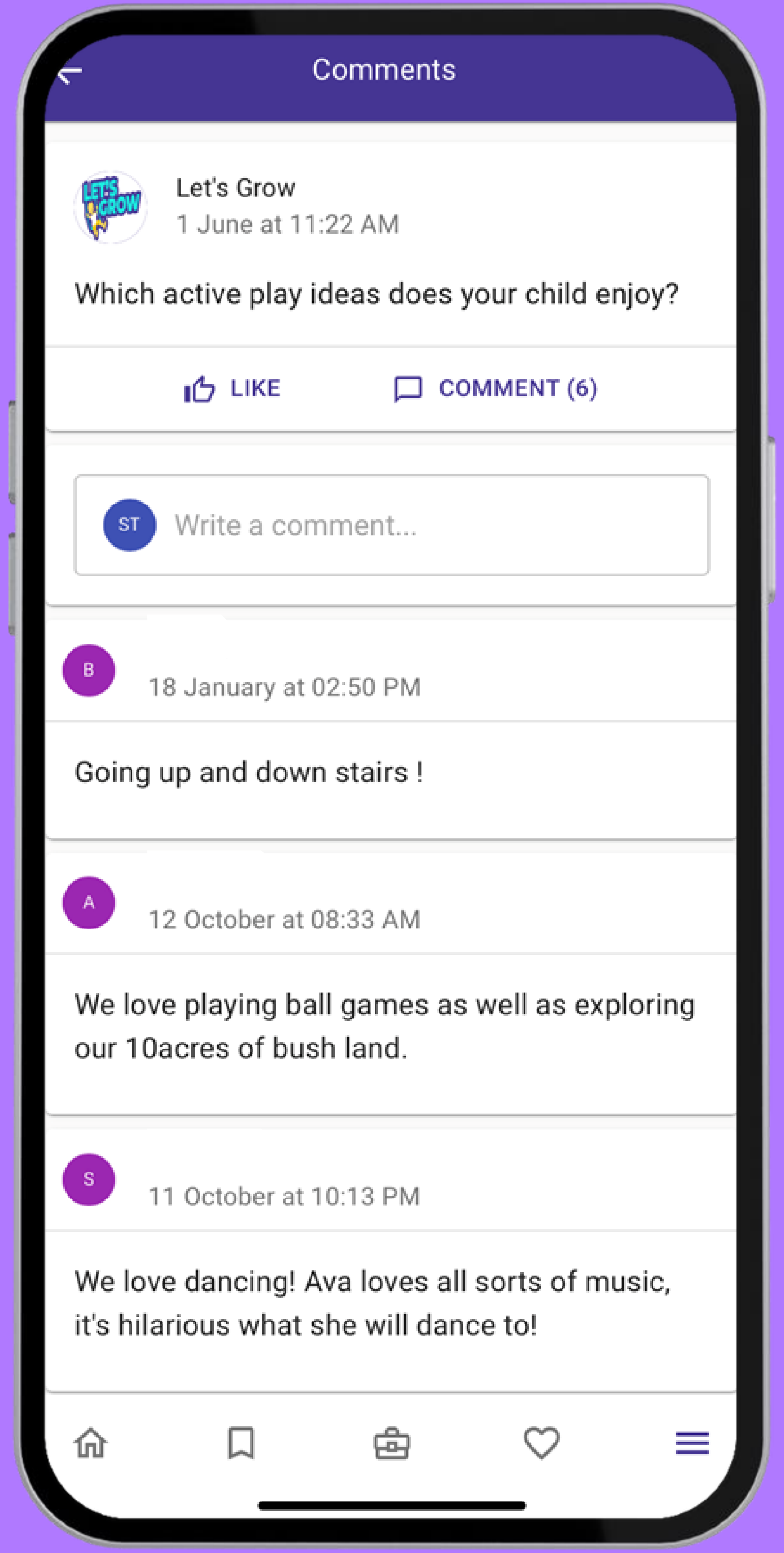

Supplement: Multimedia Appendix 1 [file mhealth_v13i1e60478_app1.pdf]
