# Supplementary material for: Parental and Demographic Predictors of Engagement in an mHealth Intervention: Observational Study From the Let’s Grow Trial
Source: JMIR Mhealth Uhealth. 2025 Jul 15;13:e60478. doi: 10.2196/60478 (PMC12308162; doi:10.2196/60478)
Supplement: Multimedia Appendix 2 [file mhealth_v13i1e60478_app2.docx]

**Table S1.** Description of the individual sub-indices included in the engagement index.

| **Index** | **Calculation** | **Description** |
| --- | --- | --- |
| Click-depth index | Mean clicks per session/total number of sessions | The average number of app pages viewed per day |
| Loyalty index | 1-(1/total number of sessions) | The number of total days with any usage data for a participant |
| Recency index | 1/mean recency^1^ | The average number of days between app use |
| Diversity index | Count of distinct features used / total number of features in the app | The number of different app features used per day, i.e., activities within modules, toolkit, community chat forum, frequently asked questions |

^1^ Reversed so higher score indicates greater engagement.
